# A comparative study of suture versus sutureless circumcision: Evaluating cosmetic outcomes, wound dehiscence and infection rates

**DOI:** 10.6026/973206300213755

**Published:** 2025-10-31

**Authors:** Ambuj Kumar Soni, Sardar Vikram Singh Bais, Sandeep Kumar, Rahul R Sha, Bhagyashree Baghel

**Affiliations:** 1 Department of General Surgery, Birsa Munda Government Medical College, Shahdol, Madhya Pradesh, India; 2 Department of Pediatrics, Birsa Munda Govermemt Medical College, Shahdol, Madhya Pradesh, India; 3 Department of Pediatrics, Shyam Shah Medical College and Associated Gandhi Memorial Hospital, Rewa, Madhya Pradesh, India; 4 Department of General surgery, JJM medical College, Devnagree, Karnataka, India; 5 Department of Pediatrics, Shyam Shah Medical College, Rewa, Madhya Pradesh, India

**Keywords:** Sutureless, circumcision, cosmetic outcomes

## Abstract

Circumcision is one of the most frequently performed surgical procedures, where optimal wound closure is crucial for healing and cosmetic outcomes. This comparative study of 50 patients evaluated isoamyl-2-cyanoacrylate tissue adhesive versus conventional suturing for circumcision wound closure. The adhesive group showed lower ASEPSIS wound scores and higher cosmetic scores on postoperative days 7 and 30 (p<0.05). Complication rates were also lower with adhesive (12%) than with sutures (20%). Isoamyl-2-cyanoacrylate proved to be an effective and cosmetically superior alternative to traditional suturing in circumcision.

## Background:

Male circumcision, the surgical removal of the foreskin (prepuce), is one of the oldest and most commonly performed surgical procedures globally [1]. Historical evidence of circumcision dates back to ancient Egyptian tombs and cave drawings [2]. According to the World Health Organization, approximately 30% of males worldwide are circumcised, with prevalence varying primarily based on religious and cultural factors [3]. In India, the incidence of circumcision in the general population is approximately 33% [4]. The procedure is typically performed during adolescence for religious, cultural, or medical reasons [5]. Various surgical techniques exist, including the dorsal slit method and sleeve resection, with local anesthesia being the preferred method of pain control [6]. Despite its commonality, circumcision is not without complications, with hemorrhage and wound infection being the most frequently reported adverse events [7]. Traditional wound closure following circumcision has involve the use of absorbable sutures, typically interrupted 3/0 chromic catgut [8]. However, this conventional approach is associated with several drawbacks, including postoperative pain, the need for suture removal, potential for track marks, and risk of needle stick injuries to healthcare workers [9]. In recent years, tissue adhesives, particularly cyanoacrylates, have emerged as a promising alternative for wound closure in various surgical procedures [10]. Cyanoacrylate tissue adhesives were first synthesized in 1949 and have since gained popularity in medical applications [11]. These adhesives polymerize upon contact with basic substances such as blood or water, forming a strong bond without requiring catalysts or solvents [12]. The latest generation of these adhesives, such as isoamyl-2-cyanoacrylate, offers improved tensile strength, flexibility, and reduced histotoxicity compared to earlier formulations [13]. Additionally, cyanoacrylates have demonstrated hemostatic and bacteriostatic properties, making them particularly suitable for wound closure in circumcision [14]. Several studies have compared tissue adhesives with traditional suturing in various surgical contexts, with many reporting advantages such as reduced procedure time, decreased postoperative pain, and improved cosmetic outcomes [15, 16-17]. However, limited research has specifically focused on circumcision, particularly in the Indian population. While some studies have suggested that tissue adhesives may offer benefits for circumcision wound closure [18, 19-20], there remains a need for comprehensive comparative studies evaluating outcomes such as wound infection, dehiscence, and cosmetic results. This research gap is particularly relevant given the increasing emphasis on patient satisfaction, cosmetic outcomes, and healthcare efficiency in modern surgical practice [21]. Furthermore, the needle-free nature of tissue adhesives offers an important safety advantage by reducing the risk of blood borne pathogen transmission to healthcare workers [22]. Therefore, it is of interest to evaluate and compare the cosmetic outcomes, wound dehiscence rates, and infection rates between the two techniques.

## Materials and Methods:

## Study design and setting:

This comparative study was conducted at the Department of General Surgery, Bapuji Hospital and Chigateri General Hospital, attached to J.J.M. Medical College, Davangere, Karnataka, India. The study period extended from November 2015 to October 2017.

## Sample size and patient selection:

A total of 50 male patients undergoing circumcision were included in the study. Patients were randomly allocated to two groups of 25 patients each: the suture group, where wound closure was performed using interrupted 3/0 chromic catgut sutures, and the adhesive group, where isoamyl-2-cyanoacrylate (Dermabond) was used for wound closure.

## Inclusion criteria:

[1] Male patients undergoing circumcision during the study period

[2] Patients willing to participate in the study and provide informed consent

[3] Patients undergoing clean elective surgical procedures

## Exclusion criteria:

[1] Patients with active infection at any site

[2] Patients with diagnosed malignancies

[3] Patients with known allergy to cyanoacrylate compounds

[4] Patients with diabetes mellitus

[5] Patients unwilling to participate in the study

## Preoperative assessment:

For all patients, a detailed history was obtained, and a thorough physical examination was performed. Routine investigations including complete blood count, blood grouping and Rh typing, random blood sugar, blood urea/serum creatinine estimation, bleeding time, clotting time, HIV, and hepatitis B surface antigen tests were conducted for all patients.

## Surgical procedure:

All circumcisions were performed using the dorsal slit method under local anesthesia. The surgical technique was standardized across both groups, with the only variation being the method of wound closure.

## Suture Group:

In the suture group (n=25), mucocutaneous approximation was achieved using interrupted 3/0 chromic catgut sutures. The sutures were placed approximately 3-4mm apart and 3-4mm from the wound edges.

## Adhesive Group:

In the adhesive group (n=25), isoamyl-2-cyanoacrylate (Dermabond) was used for wound closure. Prior to adhesive application, antibiotic ointment was applied to the urethral meatus to prevent accidental contact with the glue. Mucocutaneous approximation was achieved using a pair of toothed forceps to hold the wound edges firmly together. The adhesive was then applied in multiple thin layers (at least three) over the approximated wound edges, extending 5-10mm beyond the wound margins. Each layer was allowed to dry completely before applying the next.

## Postoperative care and assessment:

All patients were treated on an inpatient basis and received standard postoperative care. Wound healing was assessed on the 3rd, 7th, and 30th postoperative days using the ASEPSIS wound score. This scoring system evaluates wounds based on the proportion of wound involved and the presence of serous collection, erythematous changes, purulent exudates, and separation of deep tissues. The score ranges from 0 to 10, with higher scores indicating more severe wound complications. Cosmetic outcomes were evaluated on the 7th and 30th postoperative days using the Modified Hollander Cosmesis Scale. This scale assesses six clinical variables: step-off borders, edge inversion, contour irregularities, excess inflammation, wound margin separation, and overall appearance. Each variable is scored as 0 (present) or 1 (absent), with a total score of 6 considered optimal and scores of 5 or below considered suboptimal.

## Statistical analysis:

The data were analyzed using SPSS software (version 22.0). Continuous variables were expressed as mean ± standard deviation, while categorical variables were expressed as numbers and percentages. The chi-square test with Yates correction was used for univariate analysis of dichotomous variables. Student's t-test was used to compare continuous variables between the two groups. A p-value of less than 0.05 was considered statistically significant.

## Results:

A total of 50 patients were included in the study, with 25 patients in each group. The mean age in the adhesive group was 6.12 ± 2.43 years (range: 2-12 years), while the mean age in the suture group was 4.96 ± 2.44 years (range: 2-12 years). The age distribution between the two groups was comparable (p > 0.05). The ASEPSIS scores for both groups at different time intervals are presented in Table 1 (see PDF). On postoperative day 3, the mean ASEPSIS score was significantly lower in the adhesive group (0.12) compared to the suture group (0.28) (p = 0.01). The incidence of individual wound complications on day 3 is shown in Table 2 (see PDF). In the adhesive group, there were 2 cases (8%) of seroma and 1 case (4%) of erythema, amounting to a total complication rate of 12%. In the suture group, there were 3 cases (12%) of seroma and 2 cases (8%) of erythema, amounting to a total complication rate of 20%. By postoperative day 7, the mean ASEPSIS score was 0.12 in the adhesive group and 0.08 in the suture group (p = 0.003). At this time point, no complications were observed in the adhesive group, while the suture group had 1 case (4%) of seroma and 1 case (4%) of erythema, amounting to a total complication rate of 8%. By postoperative day 30, the mean ASEPSIS score was 0 in both groups, indicating complete wound healing with no complications in either group (Table 3 - see PDF, 4 - see PDF).

## Discussion:

The ASEPSIS wound scores revealed that the adhesive group had significantly better wound healing in the early postoperative period compared to the suture group. On day 3, the mean ASEPSIS score was significantly lower in the adhesive group (0.12) than in the suture group (0.28) (p = 0.01). This difference can be attributed to several factors. First, suture materials can facilitate microbial colonization and serve as a nidus for infection [23]. Second, cyanoacrylate adhesives have been shown to possess bacteriostatic properties, particularly against gram-positive cocci [24]. Third, the adhesive forms a protective barrier over the wound, potentially reducing the risk of contamination and infection [25, 26-27]. However, our results contrast with those of Cheng and Saing [28], who concluded that tissue glue has no significant advantage over suturing and that the time taken was longer in the tissue glue group. This discrepancy may be due to differences in technique or the specific adhesive used. The cosmetic outcomes assessed using the Modified Hollander Cosmesis Scale revealed a significant advantage for the adhesive group. On day 7, the mean cosmesis score was significantly higher in the adhesive group (5.84) compared to the suture group (5.72) (p = 0.04). By day 30, this difference became more pronounced, with the adhesive group achieving a mean score of 5.92 compared to 5.72 in the suture group (p = 0.002). These findings suggest that the adhesive technique provides better long-term cosmetic outcomes, which is an important consideration for patient satisfaction. The superior cosmetic outcomes with the adhesive technique can be attributed to several factors. First, the adhesive distributes tension evenly across the wound, reducing the risk of track marks and cross-hatching that can occur with sutures [29]. Second, the adhesive forms a flexible barrier that moves with the skin, potentially reducing scarring [30]. Third, the absence of suture material eliminates the inflammatory response associated with foreign bodies, which can contribute to scarring [31, 32, 33-34]. The complication rates observed in our study were lower in the adhesive group compared to the suture group. On day 3, complications were observed in 12% of patients in the adhesive group compared to 20% in the suture group. By day 7, no complications were observed in the adhesive group, while 8% of patients in the suture group still had complications. These findings suggest that the adhesive technique may be associated with fewer early postoperative complications. The lower complication rate in the adhesive group can be attributed to several factors. First, the adhesive forms a waterproof barrier that protects the wound from contamination [35]. Second, the adhesive has hemostatic properties, which may reduce the risk of hematoma formation [36]. Third, the absence of suture material eliminates the risk of suture-related complications such as granuloma formation or sinus tract development [37]. The advantages of the adhesive technique extend beyond clinical outcomes to practical considerations. The adhesive technique is faster to perform, requires no special equipment, and eliminates the need for suture removal [38-40]. Additionally, the adhesive forms a waterproof barrier that allows patients to shower earlier, potentially improving patient comfort and satisfaction [41]. These factors may contribute to reduced healthcare costs and improved efficiency [42].

## Conclusion:

We show that isoamyl-2-cyanoacrylate tissue adhesive provides an effective and reliable means of wound closure in circumcision, with outcomes comparable or superior to traditional suturing. The adhesive technique was associated with better early wound healing, fewer complications, and superior cosmetic outcomes. Additionally, the adhesive technique offers practical advantages such as faster application, elimination of suture removal, and the ability to shower earlier.
